# Second primary tumor in patients with upper aerodigestive tract cancer

**DOI:** 10.1590/S1808-86942010000200017

**Published:** 2015-10-19

**Authors:** Antonio Vitor Martins Priante, Andre Lopes Carvalho, Luiz Paulo Kowalski

**Affiliations:** MD, head and neck surgeon, Regional Hospital of the Vale do Paraíba / Taubaté University; MD, PhD, head and neck surgeon, Cancer Hospital, Barretos; MD, PhD, head of the otorhinolaryngology and head & neck surgery unit, A. C. Camargo Hospital

**Keywords:** head and neck neoplasms, neoplasms, second primary tumor

## Abstract

In the first three years after treatment of patients with squamous cell carcinoma of upper aerodigestive tract (UADT), there is a high incidence of recurrences. After the third year, the occurrence of second primary tumor (SPT) is an important cause of morbimortality.

**Aim:**

To evaluate the incidence and the characteristics of the SPT in patients with squamous cell carcinoma of UADT, treated with curative intention.

**Methods:**

Retrospective study where the incidence, localization and treatment of SPT had been analyzed and survival rates were calculated.

**Results:**

Of the 624 analyzed cases, 59 (9.4%) had SPT during follow-up (4 synchronous and 55 metachronous). The SPT free survival rate ranged from 2 to 191.3 months (median of 42.5 months). In 20 cases (33.9%) the SPT was diagnosed after the fifth year of follow-up. The most frequent site of STP was the UADT mucosa (49.1%), followed by the lungs (22.0%) and the esophagus (11.9%). The best survival after-SPT occurred in cases of UADT STP (32.2% in 5 years, median 16.2 months).

**Conclusion:**

The STP incidence was 9.4%. In 33.9% of the cases, the SPT was diagnosed after the fifth year of follow-up. The most frequent localization of STP was the UADT mucosa.

## INTRODUCTION

Head and neck squamous cell carcinomas comprise about 3% of all malignancies; they may be located in several anatomical sites of the upper aerodigestive tract.^1^ Treating these patients depends on several factors, such as experience, the tumor site and clinical stage, the patient medical status, and acceptance of therapy by patients.^2^, ^3^, ^4^

A high local and regional recurrence rate is seen within three years of treatment,^5^, ^6^, ^7^, ^8^, ^9^, ^10^ which is the main cause of treatment failure in patients with upper aerodigestive tract squamous cell carcinoma.^11^^,^^12^ The onset of a second primary tumor after three years is a significant cause or morbidity and mortality.^13^^,^^14^

Billroth as having first documented the occurrence of several simultaneous neoplasms in the same patient in 1860. Warren and Gates^15^ published in 1932 a major review of several case series of multiple primary neoplasms, and also reported 1,078 autopsies among which they found 40 cases (3.7%) of multiple tumors. In this study, the authors proposed and used the following criteria for identifying multiple primary malignancies: confirming the diagnosis of malignancy, distinguishing among each tumor, and excluding the possibility of the tumor being metastatic.

Slaughter et al.^16^ in 1953 proposed the “condemned mucosa” theory to explain the high incidence of a second primary tumor in carcinomas induced by environmental factors. These authors introduced the “field cancerization” concept to explain the occurrence of multicentre squamous cell carcinomas in the mouth.

Day and Blot^17^ gathered data from nine population registries on cancer in the United States of America and assessed the risk of a second primary tumor in 21,371 patients diagnosed with oral and pharyngeal cancer. They found a 3.7% yearly rate for the onset of a second primary tumor. Oral, pharyngeal and esophageal second primary tumors were 37% of these tumors; nose, larynx and lung tumors were 31% of these tumors; the remaining 34%, in decreasing order, were found in the lower digestive tract, the prostate, the urinary tract, the breast, and the female genital tract. The risk of developing a second primary tumor in the upper aerodigestive tract ranged from 4.2 to 30 [esophagus (RR 23.0; CI 95% 19.0 – 26.0), mouth and pharynx (RR 20.0; CI 95% 18.0 – 22.0), larynx (RR 6.8; CI 95% 5.5 – 8.4), nose and paranasal sinuses (RR 4.9; CI 95% 2.0 – 10.1), and lungs (RR 4.2; CI 95% 3.9 – 4.6)]. The risk remained high for over 5 years after the diagnosis of the primary tumor, and was higher in patients aged 60 years or less.

The purpose of this study was to assess the incidence and characterize the profile of second primary tumors in patients with head and neck squamous cell carcinomas, treated curatively at a single tertiary institution in a developing country.

## MATERIAL AND METHOD

This retrospective study consisted of a review of the medical files of all previously untreated patients with upper aerodigestive tract squamous cell carcinoma who started treatment in 1988, 1994, and 1999. These years were chosen because full data were available in the Cancer Hospital Registry, which is a reviewed and computerized database.^18^

The upper aerodigestive tract areas included in this study were:

• mouth (lips and oral cavity);
• pharynx (naso, oro, and hypopharynx);
• nose and paranasal sinuses;
• larynx.

Clinical staging of second primary tumors was reviewed based on the reported data in the files, according to the 2002 version of the American Joint Committee on Cancer (AJCC).^19^ Warren and Gates's criteria were applied in the diagnosis of second primary tumors.^15^

The date when the second primary tumor was confirmed by pathology was considered as the diagnostic date in our analysis; if this date was not available, the date when laboratory exam results were given was used. It this date was also not available, the consultation date – the medical diagnosis – was applied. Tumors diagnosed within the first six months of the diagnosis of the first tumor were classified as synchronic; after six months, they were classified as metachronic.

The SSPS 10.0 for Windows software was used for the statistical analysis.20 Descriptive statistics for absolute and relative frequencies were applied to describe the categorical variables. Central tendency measures (mean and/or median) were applied to describe quantitative variables.

The Kaplan-Meier^21^ method was applied to calculate the probability of survival; curves were compared using the log-rank test. The second primary tumor-free survival rate was given by the time in months between the beginning of treatment and the diagnostic date of the second primary tumor. The survival rate after a second primary tumor was given by the time in months between the diagnostic date of the second primary tumor and the data in which the last objective information was gathered. Significance in the statistical tests was p ≤0.05.

## RESULTS

There were 624 patients in our sample, admitted in 1988, 1994 and 1999. The majority was male (520; 83.3%). The age ranged from 10 to 93 years (median – 58.6 years). The most frequent primary tumor sites were the mouth (251 cases; 40.2%), the oropharynx (150 cases; 24.1%), and the larynx (135 cases; 21.6%) (Table 1). There were 293 clinical stage IV cases (46.9%) (Table 2).

**Table 1:** Distribution of cases according to the tumor site.

Site	number of cases (%)
Mouth	251 (40.2)
Oropharynx	150 (24.1)
Larinx	135 (21.6)
Hypopharynx	55 (8.8)
Paranasal sinus	18 (2.9)
Nasopharynx	15 (2.4)
TOTAL	624 (100.0)

**Table 2:** Clinical staging of 624 primary tumors.

Clinical stage	number of cases (%)
0	3 (0.5)
I	93 (14.9)
II	97 (15.5)
III	137 (22.0)
IV	293 (46.9)
Ignored	1 (0.2)

The follow-up period ranged from less than a month to 204.1 months (mean 49.8 months and median 28.8 months). Second primary tumors were diagnosed and recorded in 59 cases (9.4%) during follow-up. Tumors were synchronic in 4 cases (6.8%), and metachronic in 55 cases (93.2%). Three second primary tumor cases were lost to follow-up (5.1%).

**Figure 1:**
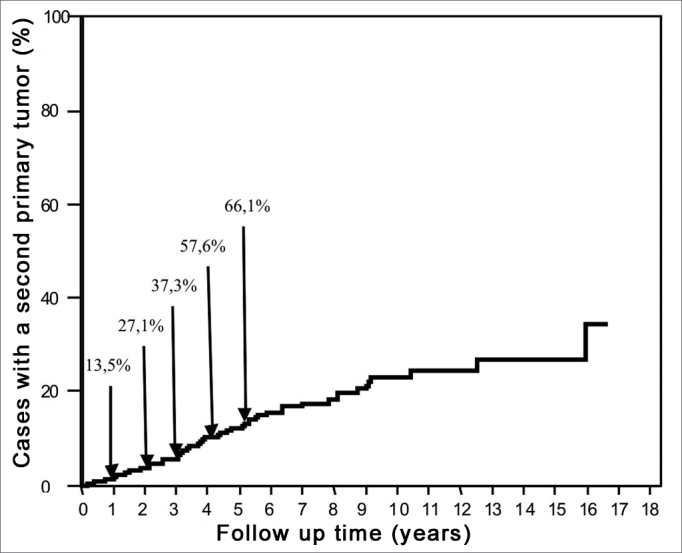
Cummulative frequency of the diagnoses of second primary tumors relative to the follow-up period in 624 cases.

The second primary tumor disease-free survival ranged from 2 to 191.3 months (mean 50.8 months and median 42.5 months). A diagnosis of a second primary tumor was made within the first follow-up year in 8 cases (13.5%). On the other hand, 20 cases (33.9%) were diagnosed after the fifth year of follow-up (Fig. 1).

The most frequent site of second primary tumors was the mucosa of the upper aerodigestive tract (29 cases, 49.1%), followed by the lung (13 cases, 22.0%), and the esophagus (7 cases, 11.9%). There were 6 cases that presented other tumors in the following sites: colon (1 case), endometrium (1 case), thyroid (1 case), prostate (1 case), kidney (1 case), and one leukemia case (Table 3).

**Table 3:** Distribution of second primary tumor cases according to the site.

Site of 2^nd^T^a^	número de casos (%)
Upper aerodigestive tract^b^	29 (49,1)
Oropharynx	14 (48,3)
Mouth	9 (31,0)
Larynx	3 (10,3)
Hypopharynx	2 (6,9)
Occult primary	1 (3,5)
Lungs	13 (22,0)
Esophagus	7 (11,9)
Stomach	4 (6,8)
Others	6 (10,2)
TOTAL	59 (100,0)

a 2^nd^T: second primary tumor

b upper aerodigestive tract

The most frequent sites of second primary tumors in the upper aerodigestive tract were the oropharynx (14 cases, 48.3%) and the mouth (9 cases, 31.0%). One case was considered as an occult second primary tumor rather than a regional recurrence, since the first tumor was a glottic in situ carcinoma that had been treated surgically. This diagnosis was made 30.4 months after treatment of the primary tumor (Table 3).

Among 59 second primary tumor cases, 12 (20.3%) were classified as clinical stage I, 9 (15.3%) were clinical stage II, 9 (15.3%) were clinical stage III, 17 (28.8%) were clinical stage IV with no distance metastases, and 8 (13.5%) had distance metastases at the time of diagnosis. Clinical staging could not be done in 4 cases (6.8%) (Table 4).

**Table 4:** Distribution of second primary tumor cases according to the clinical stage.

Clinical stage	number of cases (%)
I	12 (20.3)
II	9 (15.3)
III	9 (15.3)
IV local-regional	17 (28.8)
IV metastasis	8 (13.5)
No information	4 (6.8)
TOTAL	59 (100.0)

Radical treatment was possible in 33 cases (55.9%); surgery was the main approach. Palliative therapy was done in 16 cases (27.1%), in these cases, radiotherapy alone was the main approach (Table 5).

**Table 5:** Distribution of second primary tumor cases according to the treatment.

Variable	Category	number of cases (%)
	Radical	33 (55,9)
Treatment	Palliative	16 (27,1)
	Supportive care	10 (17,0)
	Surgery	23 (69,7)
Radical treatment	Surgery and RT^a^	4 (12,1)
	RT	4 (12,1)
	RT and CT^b^	2 (6,1)
	RT	6 (37,5)
Palliative treatment	RT e CT	5 (31,2)
	CT	3 (18,8)
	Surgery	2 (12,5)

a RT: Radiotherapy

b CT: Chemotherapy

**Figure 2:**
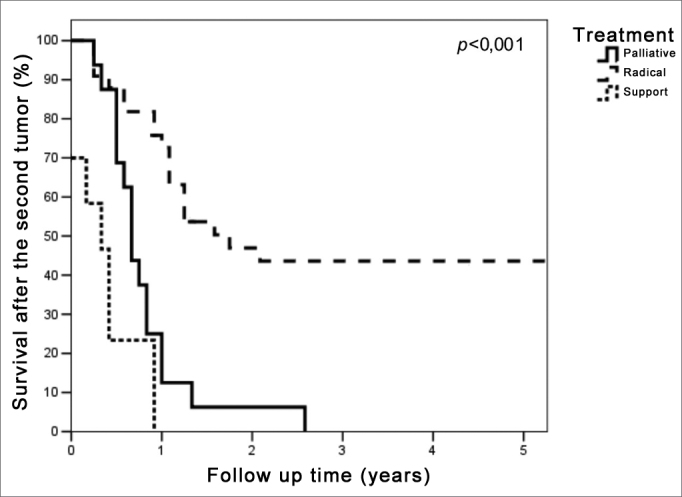
Survival curves after second primary curves according to the type of treatment of the second tumor.

The 5-year survival after a second primary tumor was significantly higher (43.5% median 21.0 months) in patients undergoing radical treatment compared to the survival in patients treated with palliative therapy (median – 8.1 months) or supportive care (median 5 months). The longest survival period in the group that received palliative care was 31.1 months; survival was 11.2 months in the group that received only supportive care (p <0.001) (Fig. 2).

**Figure 3:**
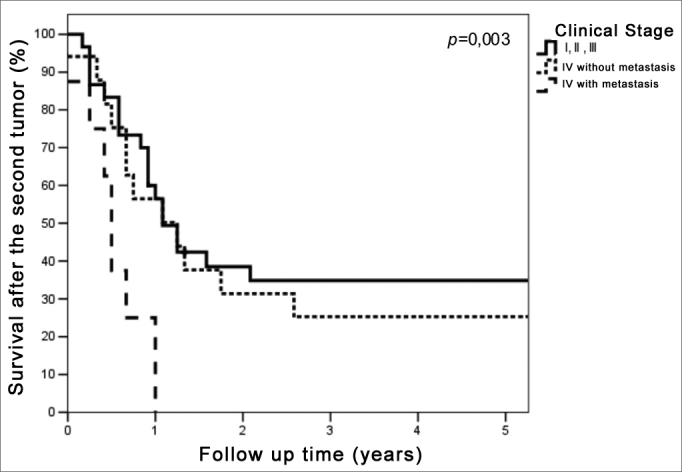
Survival curves after second primary curves according to the clinical stage of the second tumor.

The 5-year survival in patients with a clinical stage I, II or III second primary tumor was also significantly higher (34.4%, median 13.7 months) compared with the clinical stage IV group without metastases (median 15.6 months) and to the clinical stage IV with metastases group (median 6.3 months) (p=0.003) (Fig. 3). Three patients in the clinical stage IV without metastases group were alive with no evidence of active disease. All patients with upper aerodigestive tract second primary tumors (mouth, clinical stage T4N0M0; oropharynx, T4N0M0; and occult primary TxN2aM0) were followed-up for 34.2 to 46.0 months; a fourth patient with an upper aerodigestive tract second primary tumor (base of tongue T2N2bM0) was lost to follow-up after 80.2 months. The highest survival was 12.3 months in the clinical stage IV group with metastases.

**Figure 4:**
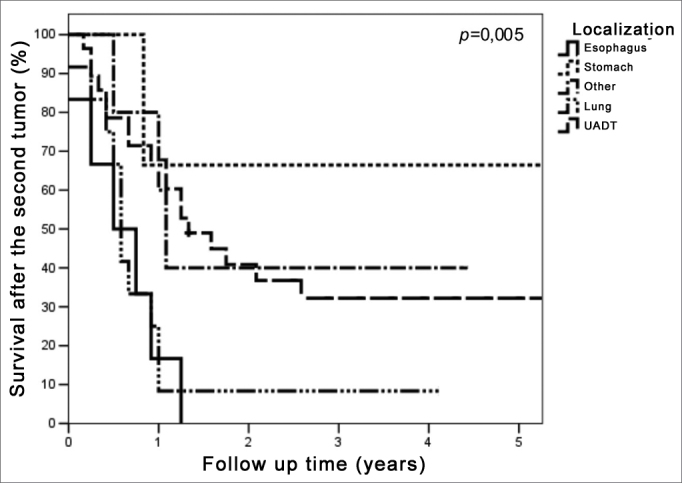
Survival curves after second primary curves according to the second tumor site.

Survival varied depending on the site of the second primary tumor (Fig. 4) (p=0.005). Only the patients with upper aerodigestive tract and stomach tumors were followed up for longer than 5 years. The 5-year survival was 32.2% (median 16.2 months) in patients with upper aerodigestive tract second primary tumors. Two patients with second primary tumors of the stomach were alive; one was followed-up for 8.4 months and received supportive care from the diagnosis of the second primary tumor, while the other was followed-up for 54.8 months, underwent radical surgery, and had no evidence of active disease. A third patient, also treated with radical surgery, had no evidence of disease after 87.6 months, but after that was lost to follow-up.

There were 13 cases with second primary tumor of the lungs; of these, only one patient remained alive, with no evidence of active disease, at 49.3 months follow-up.

This tumor had been classified as T1N0M0 clinical stage I, and was treated with surgery. The median survival in patients with second primary tumors of the lung was 7.3 months.

The longest survival in the group of patients with second primary tumors of the esophagus was 15.6 months (median 9.7 months). Among other tumors, which comprised a heterogeneous group of malignancies, one patient with a thyroid papilliferous carcinoma was alive at 51.7 months and one patient with a prostate adenocarcinoma was alive at 53.3 months follow-up.

## DISCUSSION

In this study we reviewed the files of all patients with squamous cell carcinoma of the upper aerodigestive tract that were treated at our institution in 1988, 1994 and 1999. These years were not chosen randomly. More recently, the hospital has organized a computerized database containing detailed social, demographic, and clinical data on patients (Cancer Hospital Registry). We initially we registered patients admitted in 1988, 1994,^18^ and more recently 1999, after a detailed review of the files. We thus chose these three years to make it easier to find the cases of interest for this study and to gather social, demographic, and clinical data.

Of 624 cases in this study, 59 (9.4%) developed second primary tumors. The incidence of second primary tumors in patients treated for upper aerodigestive tract tumors appears to increase in a relatively constant form with time. Vikram et al.^13^ reported a 6% yearly increase rate, where the incidence was 14.03% in his series. Day and Blot^17^ estimated that the rate of development of a second primary tumor was 3.7% a year. Our follow-up ranged from less than a month to 17 years (median 2.4 years). If we take into account our median follow-up time, the incidence of second primary tumors was 9.4%, and is placed between the two authors mentioned above.

Second primary tumors were diagnosed in 33.9% of cases after 5 years of follow-up. Di Martino et al.^22^ also found that a diagnosis of second primary tumors was made in 42% of patients after 5 years of follow-up. Franco et al.^23^ published a case control study and concluded that follow-up time is a significant factor in relation to the development of a second primary tumor.

Sturgis and Miller^14^ published a review and concluded that the risk of developing a second primary tumor is constant with time, and that these tumors are the main cause of failed treatments of upper aerodigestive tract initial stage squamous cell carcinomas. Franchin et al.^24^ studied only patients with initial laryngeal tumors treated with radiotherapy alone, and also found that the onset of a second primary tumor was the main cause of death in this group.

The main second primary tumor sites were the mucosa of the upper aerodigestive tract (49.1%), the lungs (22.0%), and the esophagus (11.9%), all of which are tumors with the same environmental risk factors. Leon et al.^25^ and Day and Blot^17^ also showed that the incidence of second primary tumors was higher in the upper aerodigestive tract. On the other hand, most of the second primary tumors in Vikram et al.'s^13^ series were in the esophagus (44%) and lungs (37.5%).

In our study, second primary tumors were classified according to the clinical stage; their biological behavior varies, however, which may compromise some comparisons. Patients with clinical stage IV upper aerodigestive tract tumors are often candidates for radical treatment, which offers significant cure rates, since patients with clinical stage IV lung tumors are metastatic.

The indication for radical treatment was significantly related with the clinical stage (I, II or III) of patients with second primary tumors (p<0.001); the time of diagnosis of a second primary tumor was unrelated with the clinical stage or survival.

The 5-year survival rate after a second primary tumor clinical stages I, II, and III, and those undergoing radical treatment, was significantly higher (p=0.003 and p<0.001).

The site of the second primary tumor also affected survival (p=0.005); the best rates occurred in patients with second primary tumor of the upper aerodigestive tract (32.2% in 5 years). It should be noted that tumors in the mucosa of the upper aerodigestive tract can be assessed locally and regionally in follow-up visits of patients with head and neck cancers, which may favor an early diagnosis.

On the other hand, the median survival time among the seven cases with a second primary tumor of the esophagus in our study was 9.7 months (maximum survival was 15.6 months). Survival was short (median 7.3 months) among the 13 cases of second primary tumors of the lung; only one patient, classified as clinical stage I, was alive after 49.3 months follow-up.

Several studies have assessed the routine use of triple endoscopy (upper digestive endoscopy, bronchoscopy, and nasofibrolaryngoscopy) in the initial evaluation of patients with head and neck tumors, with the aim of diagnosing a second primary tumor.^26^, ^27^, ^28^, ^29^, ^30^, ^31^ It is important to assess the impact of a diagnosis of second primary tumors on the survival of this group of patients by comparing them with asymptomatic patients undergoing routine follow-up not including endoscopy.

## CONCLUSION

Of 624 cases in this study, 59 (9.4%) developed second primary tumors. These tumors were synchronic in 4 cases (6.8%), and metachronic in 55 cases (93.2%).

The diagnosis of a second primary tumor was made after a 5-year follow-up period in 33.9% of cases.

The upper aerodigestive tract was most frequently affected by second primary tumors (49.2%), followed by the lungs (22.0%), and the esophagus (11.9%).

The longest survival period after second primary tumors was seen in the group of patients with second primary tumors in the upper aerodigestive tract (32.2% 5-year survival rate, median 16.2 months).
